# The Impact of mHealth Education on Changing Menstrual Hygiene Management Knowledge and Practices Among School-Going Adolescent Girls in Rural Bangladesh: A Quasi-experimental Study Protocol

**DOI:** 10.7759/cureus.52157

**Published:** 2024-01-12

**Authors:** Md Jiaur Rahman, Md Moshiur Rahman, Mohammad Habibur Rahman Sarker, Ashir Ahmed, Mohammad Ali, Md Zahidul Islam, Jesmin Ara Bubly, Delwer Hossain Hawlader, Yoko Shimpuku

**Affiliations:** 1 Health Sciences, Graduate School of Biomedical and Health Sciences, Hiroshima University, Hiroshima, JPN; 2 Nutrition and Clinical Service, International Centre for Diarrhoeal Disease Research (ICDDR), Dhaka, BGD; 3 Advanced Information Technology, Graduate School of Information Science and Electrical Engineering, Kyushu University, Fukuoka, JPN; 4 Medicine, Comilla Medical College, Comilla, BGD; 5 Global Health and Medical Science, Graduate School of Innovation and Practice for Smart Society, Hiroshima University, Hiroshima, JPN; 6 Public Health, North South University, Dhaka, BGD

**Keywords:** bangladesh, rural, adolescent, menstrual hygiene, mhealth

## Abstract

Background: Menstruation is a normal physiological process for women during their reproductive cycle, typically beginning during adolescence. During this stage, lack of knowledge, social taboos, and shyness act as barriers to proper menstrual hygiene management, rendering adolescent girls more vulnerable. This issue is highly prevalent in low- and middle-income countries. In rural areas of Bangladesh, there is a deficiency in menstrual hygiene management due to inadequate information and knowledge among adolescent girls. Therefore, this study aims to assess the effect of mHealth education on the knowledge and practices of menstrual hygiene management among school-going adolescent girls in rural Bangladesh.

Methods: This is a quasi-experimental study conducted from early June to December 2023 at a secondary high school in Chandpur, Bangladesh. Participants' data will be collected through face-to-face interviews using a structured questionnaire covering socioeconomics, knowledge of menstrual hygiene management, and practices. Pre-test data will be collected at baseline, followed by a 6-month mHealth education intervention. Afterward, post-test data will be collected using the same questionnaire. The data will be analyzed as frequency and percentage for descriptive statistics, and a paired t-test will be used to compare the pre-and post-test data.

Results: In the study, 172 participants were enrolled at baseline. Among them, 69.8% were aged 10-14 years. The outcome of this study will be published in a peer-reviewed journal. The findings will provide evidence-based information for the government, researchers, and policymakers on menstrual hygiene management using mobile health technology.

Conclusion: mHealth education can be posited as a significant tool for increasing knowledge and practices related to menstrual hygiene management in rural regions of Bangladesh.

## Introduction

Menstruation is a natural phenomenon in every woman's life during their reproductive age [1]. It is a vital stage, where a woman’s body undergoes significant reproductive changes from the onset of menstruation until menopause [2]. Menstruation can be defined as a physiological process in which tissue and blood from the uterus are shed through the female genitalia [3]. The age range of 10-19 years is considered the adolescent period, a crucial time for women [4]. During this period, they experience their first menstrual cycle, including significant emotional, physical, social, and cognitive changes. This transition from youth to puberty is challenging for girls [5]. However, societal taboos due to psychological, religious barriers, and lack of knowledge about menstruation make it more difficult. Despite being an inevitable and natural process, societal stigma and gender discrimination, along with cultural restrictions, render this critical issue silent and invisible [5]. As a result, most adolescent girls are deprived of basic information and education related to proper menstrual hygiene, exposing them to a vulnerable condition where they are forced to develop their own way of managing menstrual blood depending on the existing traditional, cultural beliefs and personal preferences [6].

Unhealthy menstrual practices particularly impact adolescent girls physically, causing dysmenorrhea, headaches, excessive bleeding, weakness, abdominal cramps, and psychologically, leading to discomfort, high stress, fear of leakage, and embarrassment about menstrual blood [7]. These issues affect their school attendance, academic progress, social involvement, and sometimes even basic activities [8]. Consequently, in later life stages, it impacts their maternal and reproductive health, increases the risk of reproductive tract infection, cervical cancer, sexually transmitted infections, and leads to poor pregnancy outcomes [9].

Bangladesh, a South Asian developing country, has a population of over 62.7 billion, with adolescents accounting for 21% of that population, as recorded by the Bangladesh Bureau of Statistics in 2015 [10]. Literature shows that poor menstrual hygiene practices among adolescent girls are more prevalent in Bangladesh, especially in rural areas [5, 11]. Factors such as inadequate knowledge and information, gender inequality, lack of sanitary products, inadequate promotion of menstrual health and hygiene, and cultural taboos contribute greatly to these issues [12]. Mothers, female relatives, and peers are the primary sources of information for young girls in Bangladesh regarding menstruation. The information provided by these older women and peers is often insufficient and incorrect, thereby affecting their menstrual management practices [5, 13]. Through health education, it is possible to increase knowledge among these individuals and positively transform their practices and attitudes [14]. Knowledge holds the power to bring about a change in attitudes and practices within a society when every individual is well-informed and aware, ultimately aiding in the practice of healthy menstrual hygiene and preventing related health issues among school-going girls [14].

In the healthcare field, eHealth utilizes Information and Communication Technology (ICT) as a supporting tool [15]. Mobile technology, a groundbreaking development of ICT, is a significant component of mHealth, playing a vital role in disseminating health-related information through mobile phones [16]. Previous studies have demonstrated that mHealth interventions delivered via mobile platforms significantly improve knowledge regarding reproductive health among various populations [17, 18, 19]. mHealth-based health education may play an important role in increasing knowledge and mitigating unhealthy menstrual practices and their associated health issues among adolescents. However, in Bangladesh, despite a high level of mobile phone usage, about 164.170 million users, specific scientific evidence is lacking on the utilization of mHealth education for improving menstrual hygiene management in rural areas [10]. Therefore, the objectives of this study are to assess the level of knowledge and practice regarding menstrual hygiene management, and the effectiveness of mHealth education interventions in improving knowledge and practice among school-going adolescent girls in rural Bangladesh.

## Materials and methods

Study design

**Figure 1:**
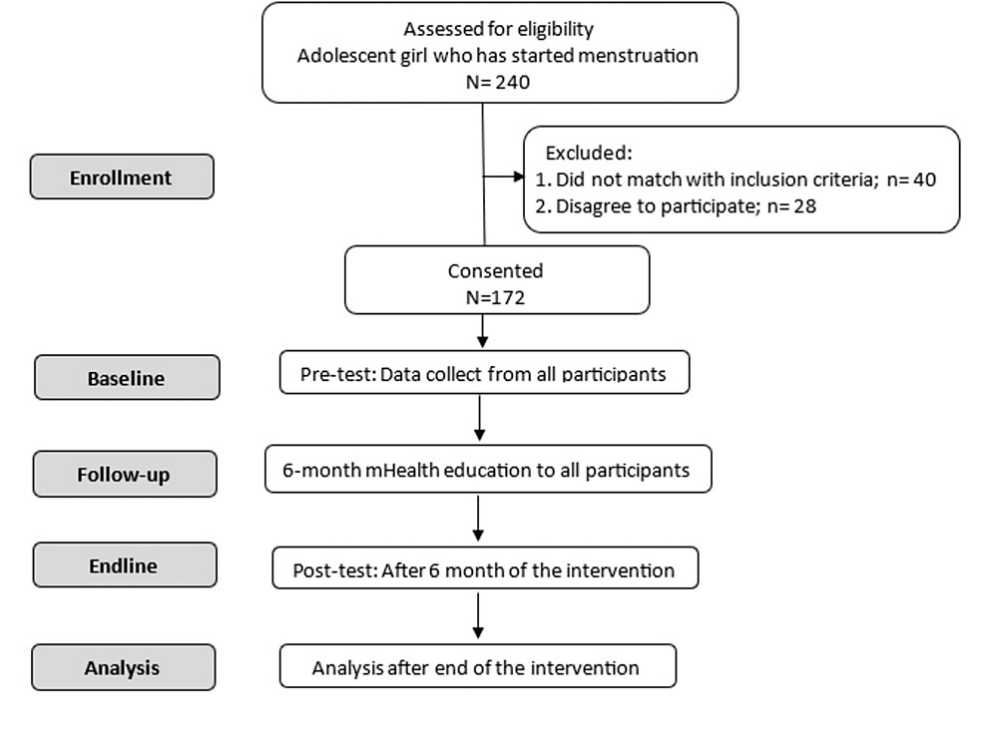
Study Flowchart

This is a school-based quasi-experimental pretest-posttest study involving adolescent girls who have begun menstruating. It is a six-month mHealth intervention that started in June 2023 and will end in December 2023. The study flowchart is depicted in Figure 1.

Study site

This study is being conducted at Baburhat High School in Chandpur Sadar Upazila, Chandpur, Bangladesh. This secondary school has 1676 students, 62% of whom are girls. The study site is located 106 km away from the capital city, Dhaka, and is part of the Chittagong division in the southern part of Bangladesh. Notably, according to the Bangladesh academic system, the secondary level encompasses classes/grades 6 to 10. The study site was purposively selected for its convenient access for researchers.

**Table 1:** Eligibility of Study Participants

Inclusion criteria	Exclusion criteria
Adolescent girl students aged between 10 and 19 years.	Those who are physically and mentally sick were not included in the study.
The current students of the study-designed school are permanent residents of the Chandpur district.	Those who are not willing to share personal information regarding menstrual hygiene.
Adolescent girls whose menstruation period has started.	Pregnant girls.
Who provides written consent to participate in the study through the legal guardian/participants?	
Those who give written informed consent through their legal guardian/participants to participate in this study.	

Sample size calculation

The estimated sample size for the study was calculated using G*Power software. With a power of 80%, a 5% significance level, and an effect size of 22.6% [20], the sample size is 156. After accounting for a 10% dropout rate due to unavailability, absence, and refusal, the final sample size is considered to be 172.

Sampling technique

A random sampling technique was employed for recruiting study participants. Information about the participants was gathered from the school register book with the assistance of a teacher. Computer-generated simple random numbers were then used to select the study participants. An external statistician, who was not involved in this study, prepared a random list, and the participation list was kept in sealed envelopes. These envelopes were stored in an office locker and were only unlocked when a study participant was ready to participate. This process was conducted under the supervision of the principal investigator, following the acquisition of written consent.

mHealth technology

A public health specialist provided two days of training for the community health care workers (CHWs) to conduct mHealth education. The training was facilitated by the public health specialist, with coordination by the principal investigator. The training session included lectures, discussions on the role of the CHWs, question-and-answer sessions, and a final discussion about the study plan. Training material was prepared to equip and ensure the CHWs acquired the necessary skills to provide health education to the target study participants.

The CHWs will provide health education using mHealth technology over mobile phone calls and Short Message Service (SMS). During the health education intervention period, the first three days of each month will feature three phone calls at 10-day intervals, and the last three days of each month will feature two phone calls at 15-day intervals during the daytime. Additionally, SMS will be sent at the same frequency and interval, five days after each phone call. The CHWs will discuss with the study participants topics such as the physiology of menstruation, hygiene practice, health risks of unhygienic menstrual practice, the use and disposal of sanitary materials, and the availability of sanitary materials. Each phone call will allow for a 10-minute discussion on menstrual hygiene management with the CHWs [Table 2]. The study will be conducted according to the following three phases.

Pre-intervention (Pretest Data Collection Phage)

The trained female community health workers (CHWs) team will collect the study data. A structured questionnaire will be used, which includes a socio-demographic section covering age, grade, marital status, living status, family structure, family income, parents' education, and parents' occupation. The knowledge section addresses physiology of menstruation, menstruation cycle, menstruation bleeding duration, regular cycle, and sanitary pad availability. The practice section explores the use of sanitary materials, genital washing during menstruation, bathing during menstruation, changing sanitary pads during the period, disposal of sanitary materials, reuse of materials, and the school environment, including the source of water, separate toilet facilities for females, lock facilities in the toilet, and private space to manage menstruation at school. Data will be collected through interviews using a structured questionnaire. This questionnaire, previously used in another study [21], will be employed in this study. Initially in English, it was later translated into the local language, Bangla. A pre-test survey will be conducted among a randomly selected five percent of study participants from the designated school for questionnaire validation. The feedback from the pre-test participants will be considered in reviewing and finalizing the questionnaire. After meeting eligibility criteria, adolescent girls will be asked to participate in the study, given written consent through their legal guardian/participants. Upon receiving written consent, the CHWs will visit the designated school to conduct face-to-face interviews.

A total of 45 questions will be asked to the participants, including eight on knowledge and eleven on practice regarding menstrual hygiene management.

Study Intervention Phase (mHealth)

A six-month mHealth education program will be provided to adolescent girls to enhance their knowledge and practice of hygiene management. A trained female CHW will deliver the health education using mobile phone calls and SMS. The content of the health education will cover the physiology of menstruation, the cause of menstruation, normal menstrual bleeding duration, menstrual cycle, availability of sanitary pads, use of sanitary pads, disposal of sanitary pads, washing genitalia during menstruation, changing sanitary materials, disposing of menstrual materials after use, and reusable cloths. Extra emphasis will be placed on encouraging open conversations about menstruation and educating themselves to reduce stigma and increase awareness, thereby promoting positive attitudes among the adolescents. Additionally, the program will share information about the complications and negative health effects of unhygienic menstrual practices during their reproductive cycle.

**Table 2:** Study Activity

	Intervention activity
Baseline	Interview the four parts of questionnaires for pre-test data collection on sociodemographics, knowledge of menstrual hygiene management, practice of hygiene management, and school facility or environmental
First 3 months	mHealth education: Mobile phone calls (10 min per call) and SMS (participants /guardians) 3 times a month by the CHWs. During the first three months, each participant will receive 9 phone calls and 9 SMS messages.
4th to 6th month	mHealth education: Mobile phone calls (10 min per call) and SMS (participants /guardians) 3 times a month by the CHWs. During the last three months, each participant will receive 6 phone calls and 6 SMS messages.
Endline	Interview the same questionnaire’s two parts for post-test data collection on knowledge of menstrual hygiene management and practice of hygiene management.

Post-intervention Phase (Post-test Data Collection)

After the six-month mHealth education program, the progress and changes in the level of knowledge and practice of menstrual hygiene management will be assessed. The effectiveness of the mHealth education will be evaluated using the same baseline questionnaire's knowledge and practice sections for the post-test data collection.

Primary outcome

The primary outcome will be the effect of mHealth education on changing the knowledge score regarding menstrual hygiene management among adolescent girls.

Secondary outcome

The secondary outcome will be the effect of mHealth education on changing the practice score regarding menstrual hygiene management among adolescent girls.

Outcome Measures

There are eight questions on knowledge and eleven questions on the practice of hygiene management. Each correct answer will be considered as indicative of good knowledge/practice and awarded a score of 1, while an incorrect answer will be considered as poor knowledge/practice and awarded a score of 0. Afterward, the mean score of each question will be compared between the pre- and post-intervention periods to assess the significance of the mHealth education effect.

Data analysis

The data will be analyzed using IBM SPSS Statistics for Windows, Version 25 (Released 2017; IBM Corp., Armonk, New York). Initially, data will be entered into Microsoft Excel 2019 software (Microsoft Corporation, Redmond, Washington) and then transferred to SPSS for analysis. A per-protocol analysis will be conducted in this study. Descriptive statistics such as frequencies, percentages, mean, and standard deviation will be calculated. The Kolmogorov-Smirnov test will be applied to check the normality of the data. Depending on the data distribution, either the Mann-Whitney U test or the Paired Sample t-test will be used to compare changes in menstrual knowledge and hygiene practices scores pre- and post-mHealth education. Statistical significance will be considered when the P value is less than 0.05.

Ethical consideration

The Institutional Review Board/Ethics Review (IRD/ERC) of North South University, Bangladesh, approved the study (reference number: 2021/OR-NSU/IRB/1102). This study is being implemented according to the Declaration of Helsinki.

Consent to Participate

All the study participants, along with their legal guardians, provided written consent for participating in the study before the baseline data collection. In the written consent, the aim of the study, along with details of the data collection and health education process, was described. Participants were informed that they could withdraw from the study at any time during the intervention period for any reason. We assured the participants that their personal information would remain confidential and that the data would be used for scientific publication without disclosing their names or any identifying information.

## Results

We recruited 172 adolescent girls at the baseline of this study, among whom 69.8% were aged 10-14 years. Of the participants, 71.5% were in grades 6-8, 97.1% were living with their parents, 22.1% had mothers with no formal education, 44.8% had fathers with no formal education, 91.9% had mothers who were housewives, 13.4% had fathers who were farmers, 80.8% received monthly pocket money from their parents, and 39.0% belonged to low-income families (Table 3).

**Table 3:** Baseline Socioeconomic Characteristics of the Study Participants (n=172)

Variable	Category	Frequency	Percentage
Age group	10–14 years	120	69.8
	15–19 years	52	30.2
Student grade	Classes 6–8	123	71.5
	Class 9	24	14.0
	Class 10	25	14.5
Living status	With parents	167	97.1
	Others	5	2.9
Mother's education	No formal education	38	22.1
	Primary education	63	36.6
	Secondary education and above	71	41.3
Mother's occupation	Housewife	158	91.9
	Working women	14	8.1
Father's education	No formal education	77	44.8
	Primary education	51	29.7
	Secondary education and above	44	25.6
Father's occupation	Farmer	23	13.4
	Business owner	50	29.1
	Job and others	99	57.6
Monthly family income (BDT)	Low income (≤10,000)	67	39.0
	Middle income (10,001–20,000)	70	40.7
	High income (≥20,001)	35	20.3
Parents provide pocket money regularly	Yes	139	80.8
	No	33	19.2

In this protocol paper, we provided the enrollment status along with the socioeconomic characteristics of the participants. After the intervention and endline data collection, we will compare the pretest and posttest data to assess the impact of mHealth education on changing the knowledge and practice regarding menstrual hygiene management. Furthermore, the study findings will be submitted to a peer-reviewed journal for publication.

## Discussion

Adolescent girls encounter various challenges in managing menstrual hygiene during their periods [22]. They have the right to be well educated and informed about healthy menstrual management before their menstruation begins. Mobile health technology offers a promising and effective means for delivering health education, facilitating positive knowledge acquisition, behavior modification, and cultural sensitivity, especially in rural communities [16]. To our best knowledge, this is the first study utilizing mHealth technology to improve knowledge and practice regarding menstrual hygiene management among school-going adolescent girls in the rural areas of Bangladesh. Additionally, a school-based intervention study in an urban area of Bangladesh has shown that face-to-face health education effectively increased menstrual hygiene knowledge and practices among adolescent girls [7]. Utilizing mobile phones for health education can be a motivating approach to enhance awareness and knowledge about hygiene management practices among the participants. Beyond the acquisition of knowledge, mHealth education plays a pivotal role in empowering adolescent girls to effectively manage their menstrual hygiene. This knowledge helps to break down social taboos and stigmas associated with menstruation, thereby boosting their confidence. Such education is crucial in fostering a positive attitude towards their menstrual health, which contributes to their overall well-being. We anticipate that our mHealth program will be effective for menstrual hygiene management among school-going adolescent girls.

Limitation

A limitation of our study is that we randomly selected participants from a secondary school in Chandpur district, Bangladesh, which will not represent the menstrual hygiene management of the entire rural school-going adolescent girl. Also, it is a quasi-experimental design that cannot fully control confounding factors that may affect the appropriate finding.

## Conclusions

Research findings may demonstrate the effectiveness of mHealth education and could lead to recommendations for implementing such health education at the national level using phone calls and SMS. mHealth can serve as an effective tool to increase knowledge and promote proper menstrual hygiene management practices among school-going adolescent girls. Additionally, mHealth interventions have the potential to enhance their understanding and promote their overall health and well-being.
